# How can We Tackle Energy Efficiency in IoT Based Smart Buildings?

**DOI:** 10.3390/s140609582

**Published:** 2014-05-30

**Authors:** M. Victoria Moreno, Benito Úbeda, Antonio F. Skarmeta, Miguel A. Zamora

**Affiliations:** Department of Information and Communications Engineering, University of Murcia, 30100 Murcia, Spain; E-Mails: bubeda@um.es (B.Ú.); skarmeta@um.es (A.F.S.); mzamora@um.es (M.A.Z.)

**Keywords:** internet of things, smart building, energy efficiency

## Abstract

Nowadays, buildings are increasingly expected to meet higher and more complex performance requirements. Among these requirements, energy efficiency is recognized as an international goal to promote energy sustainability of the planet. Different approaches have been adopted to address this goal, the most recent relating consumption patterns with human occupancy. In this work, we analyze what are the main parameters that should be considered to be included in any building energy management. The goal of this analysis is to help designers to select the most relevant parameters to control the energy consumption of buildings according to their context, selecting them as input data of the management system. Following this approach, we select three reference smart buildings with different contexts, and where our automation platform for energy monitoring is deployed. We carry out some experiments in these buildings to demonstrate the influence of the parameters identified as relevant in the energy consumption of the buildings. Then, in two of these buildings are applied different control strategies to save electrical energy. We describe the experiments performed and analyze the results. The first stages of this evaluation have already resulted in energy savings of about 23% in a real scenario.

## 1. Introduction

Buildings, both residential and commercial, represent one of the highest energy consumption fields in the world. This tendency is particularly pronounced in developed countries, where between 20% and 40% of the total energy consumed is related with buildings [1]. Reduction of the carbon footprint on a global scale as well as ensuring energy efficiency of buildings are key goals of high priority for multi-disciplinary researchers in the fields of building engineering and energy policy. International actions to improve energy efficiency in buildings have already been proposed. From European Commission, for instance, a recast of the Directive about “*Energy Performance of Buildings*” (2010/31/EU) [2] was issued few years ago. This Directive proposed the adoption of measures to improve the performance of appliances located in buildings, including lighting and, especially, boilers and ventilation systems, in an attempt to reduce their associated energy consumption.

In order to be able to reduce the amount of energy consumed by improving the efficiency of the supply systems, a crucial step is to analyze how energy is currently consumed in buildings. Given the increasing demands being placed upon heating, ventilation and air conditioning (HVAC) systems to provide thermal comfort, there is a clear need to address one of the underlying drivers of energy consumption. Standardization organizations are also aware of this concern [3], such as the International Organization for Standardization (ISO), which has set up the technical committees ISO/TC 163, “*Thermal Performance and Energy Use in the Built Environment*”, and ISO/TC 205 “*Building Environment Design*”. Across them, these groups recognize that, apart from the physical architecture of a building, intelligent and automated systems are needed to improve comfort and energy efficiency, as is stated, for example, in ISO 16484 proposal, “*Building Automation and Control Systems*”.

Analysis of the energy efficiency of the built environment has received growing attention in the last decade [4–6]. Various approaches have addressed energy efficiency of buildings using predictive modeling of energy consumption based on usage profile, climate data and building characteristics. On the other hand, studies about the impact of displaying public information to occupants are shown useful to modify their individual behavior in order to obtain energy savings [7,8]. Nevertheless, most of the approaches proposed to date only provide partial solutions to the overall problem of energy efficiency in buildings, where different factors are involved in a holistic way, but until now have been addressed separately or even neglected by previous proposals. This division is frequently due to the uncertainty and lack of data and inputs included in the management processes, so that analysis of how energy in buildings is consumed is incomplete. In other words, a more integral vision is required to provide accurate models of the energy consumed in buildings [9]. Therefore, there is a lack of analysis to indicate the steps required to find solutions to energy efficiency in buildings.

The need for robust characterization of energy use in buildings has gained attention in light of the growing number of projects and developments addressing this topic. Bearing in mind that buildings with different functionalities have different energy use profiles, it is necessary to carry out an initial characterization of the main contributors to their energy use. For instance, in residential buildings the energy consumed is mainly due to the indoor services provided to their occupants (associated to comfort), whereas in industrial buildings energy consumption is associated mostly to the operation of industrial machinery and infrastructures dedicated to production processes.

In this context, the integration and development of systems based on Information and Communication Technologies (ICT) and, more specifically, the Internet of Things (IoT) [10], are important enablers of a broad range of applications, both for industries and the general population, helping make smart buildings a reality. IoT permits the interaction between smart things and the effective integration of real world information and knowledge in the digital world. Smart (mobile) things endowed with sensing and interaction capabilities or identification technologies (such as RFID) provide the means to capture information about the real world in much more detail than ever before.

In this respect, there is a huge opportunity to improve the most competitive actors to offer more cost-effective, user-friendly, healthy and safe products for buildings. In Europe, for instance, the area of energy management systems in buildings has only just started, but is rapidly moving towards a technology-driven status with rising productivity. This is mainly due to the need to reduce energy and greenhouse gas (GHG) in line with the EU 2020 and 2050 objectives [11]. This will ultimately create a solid foundation for continuous innovation in the building sector through sustainable partnerships, fostering an innovative eco-system as a fundamental corner-stone for smart cities.

In this work, we talk about what are the main drivers of the energy consumed in buildings, and analyze what are the main parameters that should be considered to be included in any building energy management. The goal of this analysis is to select the most relevant parameters to control the energy consumption of buildings according to their context, selecting them as input data of the management system. With the aim of validating our approach to achieve significant energy savings, we carry out different experiments following this approach, which demonstrate the need to consider holistic solutions to the problem of energy efficiency in buildings. For this, we select three reference smart buildings, and where our automation platform for energy monitoring is deployed. We carry out some experiments in these buildings to demonstrate the influence of the parameters identified as relevant in the energy consumption of the buildings. Then, in two of these buildings are applied different control strategies to save electrical energy. We describe the experiments performed and analyze the results. The first stages of this evaluation have already resulted in energy savings of about 23%.

The rest of the paper is organized as follows: Section 2 reviews previous solutions presented in the literature to the problem of energy efficiency in buildings. Section 3 makes a general description of the problem of optimizing energy consumption in buildings, and analyze the main parameters identified as relevant for inclusion in any building management system to save energy. Section 4 describes our proposal of a smart building based on an automation platform in charge of monitoring, managing and controlling the building infrastructures, and explains our strategy of optimum energy management. Section 5 presents some experiments carried out in three buildings used as reference. For this, Section 5.1 describes the deployments carried out in these buildings, and Section 5.2 details the experiments performed and which shows the suitability of our solution when energy savings are to be achieved. Finally, Section 6 concludes the paper and presents possible future lines of work.

## 2. Related Work

Although much interest has been put into smart building technologies, the research area of using real-time information has not been fully exploited yet. In order to obtain an accurate simulation model, a detailed representation of the building structure and the subsystems is required, although it is the integration of all systems that requires the most significant effort. Initial solutions to energy efficiency in buildings were mainly focused on non-deterministic models based on simulations. A number of simulation tools are available with varying capabilities. In [12] or [13] a comprehensive comparison of existing simulation tools are provided. Among these tools are ESP-r [14] or Energy Plus [15]. However, this type of approach relies on very complex predictive models based on static perceptions of the environment. For example, a multi-criteria decision model to evaluate the whole lifecycle of a building is presented in [16]. The authors tackle the problem from a multi-objective optimization viewpoint, and conclude that finding an optimal solution is unreal, and that only an approximation feasible.

With the incessant progress of ICT and sensor networks, new applications to improving energy efficiency are constantly emerging. For instance, in office spaces, timers and motion sensors provide a useful tool to detect and respond to occupants while providing them with feedback information to encourage behavioral changes. The solutions based on these approaches are aimed at providing models based on real data sensor and contextual information. Intelligent monitoring systems, such as automated lighting systems, have limitations such as those identified in [17], in which the time delay between the response of these automated systems and the actions performed can reduce energy savings, whilst an excessively fast response could produce inefficient actions. These monitoring systems, while contributing towards energy efficiency, require significant investment in intelligent infrastructure that combines sensors and actuators to control and modify the overall energy consumption. The cost and difficulty involved in deploying such networks often constrain their viability. Clearly, an infrastructureless system that uses existing technology would provide a cheaper alternative to building energy management. On the other hand, building energy management must face up the inaccuracy of sensors, the lack of adequate models for many processes and the non-deterministic aspects of human behavior.

In this sense, there is an important research area that proposes to implement artificial intelligence techniques to process all data related with the problem, and as a way of providing intelligent building management systems solving the above drawbacks. This approach involves models based on a combination of real data and predictive patterns that represent the evolution of the parameters affecting the energy consumption of buildings. An example of such an approach is [18], in which the authors propose an intelligent system able to manage the main comfort services provided in the context of a smart building, *i.e.*, HVAC and lighting, while user preferences concerning comfort conditions are established according to the occupants' locations. Nevertheless, the authors only propose the inputs of temperature and lighting in order to make decisions, while many more factors are really involved in energy consumption and should be included to provide an optimal and more complete solution to the problem of energy efficiency in buildings. Furthermore, no automation platform is proposed as part of the solution.

On the other hand, and regarding building automation systems, many works extend the domotics field which was originally used only for residential buildings. A relevant example is the proposal given in [19], where the authors describe an automation system for smart homes based on a sensor network. However, the system proposed lacks automation flexibility, since each node of the network offers limited I/O capabilities through digital lines, *i.e.*, there is no friendly local interface for users, and most importantly, integration with energy efficiency capabilities is weak. The work presented in [20] is based on a sensor network to cope with the building automation problem for control and monitoring purposes. It provides the means for open standard manufacturer-independent communication between different sensors and actuators, and appliances can interact with each other with defined messages and functions. Nevertheless, the authors do not propose a control application to improve energy efficiency, security or living conditions in buildings.

The number of works concerning energy efficiency in buildings using automation platforms is more limited. In [21], for instance, a reference implementation of an energy consumption framework is provided, but it only analyzes the efficiency of ventilation system. In [22] the deployment of a common client/server architecture focused on monitoring energy consumption is described, but without performing any control action. A similar proposal is given in [23], with the main difference that it is less focused on efficiency indexes, and more on cheap practical devices to cope with a broad pilot deployment to collect the feedback from users and address future improvements for the system.

In this work we present a solution that involves collecting and analyzing information from heterogeneous sources, and propose concrete actions to minimize energy consumption considering the specific context of the target building. For that, we propose a platform based on the optimal integration and use of gathered information, which is provided by, among others, the users themselves. This generic and interoperable smart building automation proposal addresses the problem of energy efficiency of buildings, comfort services for occupants, environmental monitoring and security issues, among others. It uses a flexible IoT approach, which allows data to be gathered from a plethora of different sources, and is able to control a wide range of automated appliances in the building. Thus, our smart energy building management analyzes all monitored data provided by automated devices and, depending on the required operation mode and considering the energy balance status of the building, takes real-time decisions to improve energy efficiency, while retaining conditions at different user-acceptable comfort levels.

In the next section we review different aspects that need to be analyzed before a solution can be proposed to save energy in buildings. Additionally, we describe the steps that are essential for providing optimum solutions that address energy efficiency in buildings based both on real data sensors and data prediction.

## 3. Towards Smart Buildings: Optimization of Energy Efficiency

Optimizing energy efficiency in buildings is an integrated task that comprises the whole lifecycle of the building. According to the literature [21], the main stages are:

- Design, using simulations to predict the energy performance.
- Construction, testing individual subsystems.
- Operation, monitoring the building and controlling actuators.
- Maintenance, solving infrastructure problems due to energy deficiencies.
- Demolition, recycling materials and usable elements.

During these phases, it is necessary to continuously re-engineer the indexes that measure energy efficiency to adapt the energy management system to the building's conditions. If we take as reference the energy performance model for buildings proposed by the *CEN Standard EN15251* [24], it proposes criteria for dimensioning the energy management of buildings, while indoor environmental requirements are maintained. According to this standard, there are static and dynamic conditions that affect energy consumption of buildings. Given each building has a different static model according to its design, we try to provide a solution for energy efficiency focusing on analyzing how dynamic conditions affect energy consumed in buildings. Thus, we propose an initiative for the challenges involved in the living stage of buildings *Performance monitoring and management* from the before list. In this stage, we need to identify what are the main drivers of energy use in buildings. Because after monitoring these parameters and analyze the energy consumed associated to them, we can model the impact of each one in the energy consumption, and then, propose strategies of control that let save energy in the target building. The main idea of this approach is to provide anticipated responses to ensure energy efficiency in buildings.

Bearing in mind all these concerns, following we describe the stages [25] that must be carried out to realize energy-efficient buildings.

### 3.1. Monitoring

During monitoring phase, information from heterogeneous sources is collected and analyzed before proposing concrete actions to minimize energy consumption considering the specific context of buildings. Bearing in mind that buildings with different functionalities have different energy use profiles, it is necessary to carry out an initial characterization of the main contributors to their energy use. For instance, in residential buildings the energy consumed is mainly due to the indoor services provided to their occupants (associated to comfort), whereas in industrial buildings energy consumption is associated mostly to the operation of industrial machinery and infrastructures dedicated to production processes. Considering this, and taking into account the models for predicting the comfort response of buildings occupants given by the *ASHRAE* [26], we describe below the main parameters that must be monitored and analyzed before implementing optimum energy building managements. In this way, from this set of parameters affecting energy consumption in buildings, we can extract the input data to include in the proposal of solution.

1. **Electrical devices always connected to the electrical network**. In buildings, it is necessary to characterize the minimum value of energy consumption due to electrical devices that are always connected to the electrical network, since this represents a constant contribution to the total energy consumption of the building. For this, it is necessary to monitor over a period of time the energy consumed in the building when there is no other contributor to the total energy consumed. This value will be included as an input to the final system responsible for estimating the daily electrical consumption of the building.
2. **Electrical devices occasionally connected.** Depending on the kind of building under analysis, different electrical devices may be used with different purposes. For instance, for productive aims in a company, for providing comfort in a home, *etc*. On the other hand, the operation of such devices could be independent of the participation and behavior of the occupants; for example, in the context of a factory or an office where there are schedules and rules. Whatever the case, a recognition of the operation pattern of devices must be included in the final system responsible for estimating the daily electrical consumption of the building. To obtain these patterns it is necessary to monitor previously the associated energy consumption of every device or appliance. To monitor each component separately in the total power consumption of a household or an industrial site over time, cost effective and readily available solutions include Non-Intrusive Load Monitoring (NILM) techniques [27].
3. **Occupants' behavior**. Energy consumption of buildings due to the behavior of their occupants is one of the most critical point in every building energy management. This is mainly because occupant behavior is difficult to be characterized and controlled due to its uncertainty and dynamic. First of all, it is necessary to have solved the occupants' localization before behavior models associated to them can be provided. Depending on the building context, the impact of occupants behavior on the total energy consumption is different. For example, in residential buildings the impact of occupants behavior in the energy consumed is one of the greatest, followed by environmental conditions. However, in buildings with productive goals, the electrical consumption due to the appliances and devices working for such goals is usually the main contributor to the total energy consumed in the building. Therefore, it is required to monitor and analyze this issue to be able to provide behavior patterns that will be included in the final estimation of the daily energy consumption of the building. Occupants' behavior can be characterized for features such as:
  - Occupants localization data
  - Activity level of occupants
  - Comfort preferences of occupants
4. **Environmental conditions**. Parameters like temperature, humidity, pressure, natural lighting, *etc.* have a direct impact on the energy consumption of buildings. Nevertheless, depending on the specific context of the building and its requirements, this impact will differ and be greatest in the case of indoor comfort services (like thermal and visual comfort). Therefore, forecasts of the environmental condition should also be considered as input for the final estimation of energy consumption of the building.
5. **Information about the energy generated**. Sometimes, alternative energy sources can be used to balance the energy consumption of the building. Information about the amount of daily energy generated and its associated contextual features can be used to estimate the total energy generated daily. This information allows us to design optimal energy distribution or/and strategies of consumption to ensure the energy-efficient performance of the building.
6. **Information about total energy consumption**. Knowing the real value of the energy consumed hourly or even daily permits the performance and accuracy of the energy building management to be evaluated, and make it possible to identify and adjust the system in case of any deviation between the energy consumption predicted and the real value. Besides, providing occupants with this information is crucial, making them aware of the energy that they are using at any time, and encouraging them to make their behavior more responsible.

### 3.2. Information Management

An intelligent management system must provide proper adaptation countermeasures for both automated devices and users, with the aim of satisfying the most important services provided in buildings (comfort) and energy efficiency requirements. Therefore, energy savings need to be addressed by establishing a tradeoff between the quality of services provided in buildings and the energy resources required for the same, as well as its associated cost.

### 3.3. Automation System

Automation systems in buildings take inputs from the sensors installed in corridors and rooms (light, temperature, humidity, *etc.*), and use these data to control certain subsystems such as HVAC, lighting or security. These and more extended services can be offered intelligently to save energy, taking into account environmental parameters and the location of occupants. Therefore, automation systems are essential to answer the needs for monitoring and controlling with energy efficiency requirements [28].

### 3.4. Feedback and User Involvement

Feedback on consumption is necessary for energy savings and should be used as a learning tool. Analysis of smart metering, which provides real-time feedback on domestic energy consumption, shows that energy monitoring technologies can help reduce energy consumption by 5% to 15% [7]. As can be deducted, a set of subsystems should be able to provide consumption information in an effective way. These subsystems are: electrical lighting, boilers, heating/cooling systems, electrical panels, *etc*.

On the other hand, to date, information in real-time about building energy consumption has been largely invisible to millions of users, who had to settle with traditional energy bills. In this, there is a huge opportunity to improve the offer of cost-effective, user-friendly, healthy and safe products for smart buildings, which provide users with increased awareness (mainly concerning the energy they consume), and permit them to be an input of the underlying processes of the system. Therefore, an essential part of any intelligent management system is users involvement, through their interactions and their associated data (identity, location and activity), so that customized services can be provided.

## 4. City Explorer: A Holistic Platform for Smart Buildings

A smart building provides occupants with customized services thanks to the intelligence of their contained objects, be it an office, a home, an industrial plant, or a leisure environment. Since the building environment affects the quality of life and work of all citizens, buildings must be able of not only providing mechanisms to minimize their energy consumption (for instance, integrating their own energy sources to ensure their energy sustainability), but also of improving habitability and productivity.

Sensor and actuator deployments in buildings need to be optimized in such a way that the associated cost is offset by the economic value of the energy saving. Note that monitoring the whole area of large buildings is not feasible nor realistic. Moreover, to the behavior patterns obtained after data monitoring, real sensor data about such inputs should be considered in the final energy management system. In this way, the system is able to adapt itself to changes in the building context as well as to new situations not included in the initial models.

On the other hand, it is possible to make a spatial division of the building according to the energy consumption of each area, so that different levels of monitoring and management can be applied. For example, while denser sensor deployments may be required to provide data about energy consumption in some zones of the building, in others, very few sensors or even none may be necessary when the energy consumption of these areas is negligible compared with the total.

Following this approach, an optimum design for the building energy management can be provided by: (i) considering many data sources and the characterization of the building context; (ii) applying appropriate techniques of data fusion and intelligent data processing to take decisions to save energy in an efficient way.

In this section we present our core platform for smart buildings, and describe our proposal for energy building management, which includes as input data the variables mentioned above as relevant in the energy consumption of buildings.

The automation platform integrated in our smart building proposal is based on the *City explorer* system, whose main components are described in details in [29]. This automation platform composes all internal equipment installed in the building and all the external connectivity infrastructure required to provide remote access, technical tele-assistance, security and energy efficiency/comfort services.

**Figure 1.**
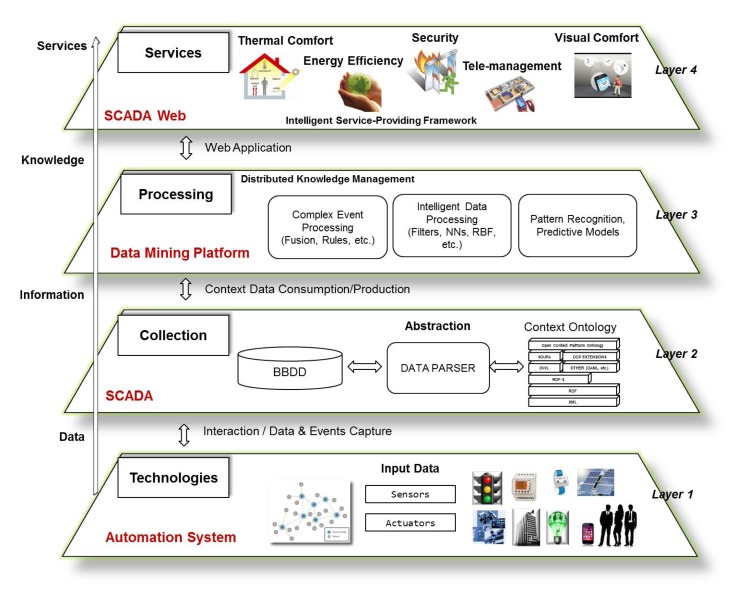
Layers of the base architecture of our smart building management system.

The architecture of this platform is modeled in layers, which are generic enough to cover the requirements of different smart environments, as addressed in the context of smart buildings. Figure 1 shows the layers structure which is fully detailed in the previous work [30]. Basically, it consists of a first layer of data sensing able to manage multiple data sources as well as heterogeneous technologies. The second layer is dedicated to data processing to convert all collected data into a common format. To do this, some relevant works on energy efficiency with building automation systems employing semantic technologies can be found in [31,32]. Nevertheless, in this work the semantic perspective is not the main goal up to this moment for the data processing in our proposed system, although we consider this as a key and very critical aspect from an architecture point of view, and certainly it is in our next step of the evolution of our platform. Thus, currently we are implementing a common language format to represent all data sensed by sensors deployed in buildings and available through City explorer, taking as reference the ontologies already proposed in the literature. The next layer consists of applying data processing techniques and intelligent rules according to the final application or service selected for each specific building context (a home, an office, an factory, *etc.*). In this way, for example, a mechanism for solving the indoor localization problem has been implemented. This mechanism is explained with detail in the previous work with reference [33]. As a summarize, the main techniques implemented for this mechanism are based on an estimator based on Radial Basis Function Networks (RBF) and a tracking technique based on a Particle Filter (PF). Another example of data processing technique implemented is an optimization technique based on Genetic Algorithm in charge of providing optimal comfort conditions to the occupants of buildings. In the previous work with reference [30] more details about the mining data algorithms implemented in this layer are described. Finally, as can be seen in the last layer depicted in Figure 1, services such as thermal comfort, security, tele-management, energy efficiency, *etc.* can be provided in the context of smart buildings.

The City explorer system is composed of two main subsystems: a network of Home Automation Modules (HAMs) and the Supervisory Control and Data Acquisition system (SCADA). Each HAM is an embedded system based on a low consumption CPU (32bits 4 MB) and connected to all the appliances, sensors and actuators installed in the building. These devices centralize the intelligence of each space, controlling the configuration of the installed appliances. The HAM includes an optional human-machine interface (HMI). In addition, several control panels can be spread throughout the building to control specific parts. These include an embedded solution with an HMI adapted to the controlled devices. For example, in a three-story office building, each floor could have a control panel set to automatically open windows, turn the air conditioning to the desired temperature, or open and close the blinds according to the desired light intensity before using artificial lighting. These examples are developed case studies that diminish the power consumption and contribute to environmental preservation. The local gateway offers value-added services for managing and monitoring tasks, but it does not directly control appliances or actuators. Instead, this gateway communicates with the HAM using a UDP-based protocol.

**Figure 2.**
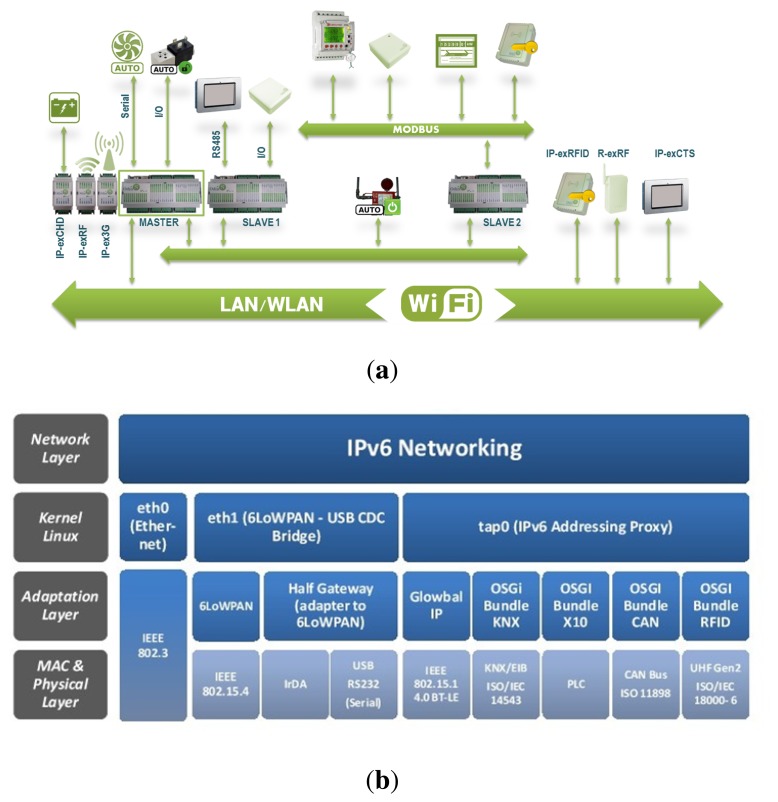
Schemas of integration in City explorer. (**a**) Schema of components integration in City explorer platform; (**b**) Schema of technologies integration in City explorer platform.

City explorer bets on current specifications to connect the HAM with appliances and the remaining devices, and it proposes a novel communication protocol that connects the architecture's IP-based elements through UDP. IP-based elements are considered the local gateway; control panels and architectural elements outside the building are the remote gateway. Sensors and actuators can be self-configured and controlled remotely through the Internet, enabling a variety of monitoring and/or control applications. Each HAM of City explorer supports most extended communication protocols, enabling it to connect with a wide range of external devices. In addition to wired buses, wireless IP connectivity is provided by standards such as ZigBee (or 6LowPAN) and Bluetooth in order to allow an IoT approach to connect SCADA and all sensors and actuators. Controller Area Network (CAN) bus may also be used to extend the operation range or provide a more distributed wiring solution. X-10 connections over the power line (PLC) are also available for low-cost domotic installations, whereas the KNX-EIB controller offers a powerful solution for connecting with more complex appliances. Devices based on the RS-485 protocol can be connected, and the Modbus protocol is also supported. In Figure 2a a schema showing the integration of different components in the City explorer platform is depicted, and in Figure 2b a schema of the technologies integration is showed.

We use the OSGi (Open Services Gateway initiative) framework in the gateway to manage the life cycle of services that cover these features. Thus, a service that implements the underlying UDP protocol to connect with the HAM enables the implementation of more complex applications; and the HTTP service that the OSGi framework offers is used by a Web application to provide local and remote management capabilities through a 3D interface. In addition, the building owner can also use an SMS-based remote control strategy if the wired Internet access is out of service.

A SCADA platform acts a as gateway to offers value-added services for management and monitoring, but it is not in charge of performing any control over appliances or actuators directly. Instead, this gateway communicates with the HAM using a UDP-based protocol later explained. This SCADA will be usually provided remotely in a high-end server. Some other solutions leave these control actions to a local PC-based gateway, which is understood as a not appropriate strategy. A SCADA-based solution is used in City explorer to give extra services to inhabitants, and perform networking tasks from the transport to the application layer in the OSI stack.

**Figure 3.**
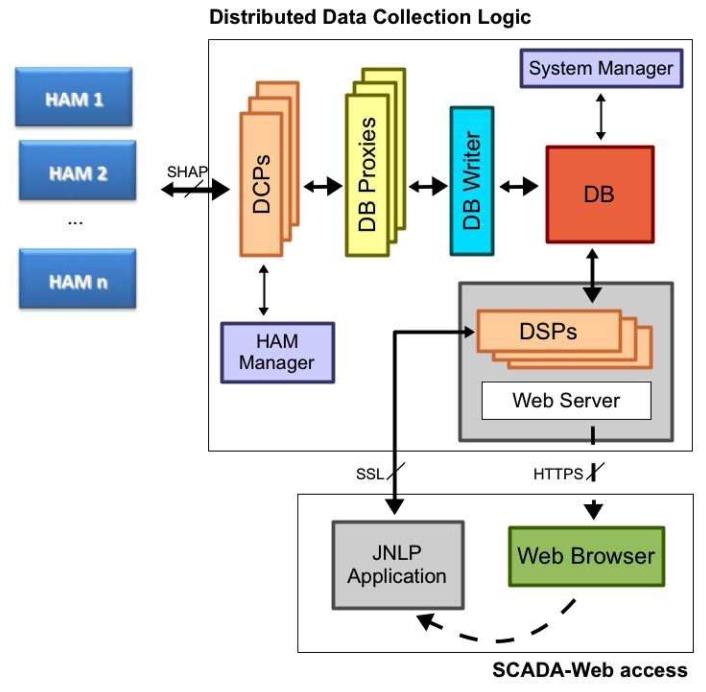
Architecture of the SCADA distributed logic.

A remote data and request processing management system is proposed. The SCADA has been designed on the basics of a distributed data collection logic. It collects building data, sensor measurements and energy efficiency information from buildings in a reliable way, and provides processed information to users/administrators through a SCADA access. Its architecture is shown in Figure 3. As can be seen, data from HAMs is collected by a set of Data Collection Points (DCPs) by means of the SHAP protocol. HAMs choose one of these DCPs according to the observed performance and an initial priority list. All data collected by DCPs is then sent to Data Base Proxies, in charge of turning HAM measurements into data records. Several Data Base Proxies provide reliability to the system for accessing the database. Finally, an intermediate stage for providing a buffered and synchronized access to the database is provided by DB Writer. All this information flow provides a fault-tolerant design against eventual problems in the different modules.

As can be seen in Figure 3, two management modules have been included in the data collection system: HAM Manager and System Manager. HAM Manager is used to keep track of all building connections, and it enables administrators to check the HAM firmware. System Manager is an always-on service that monitors the operation of all modules. It periodically reads status information of all modules (DCPs, DB Proxies, *etc.*) from the database, since each new record also includes status stamps of each system module.

All collected information from HAMs is finally provided to users/administrators (and the rest of building-external entities) through a SCADA access (called SCADA-Web). This is also illustrated in Figure 3. By means of a PC platform, a common Web Browser can be used to access a URL of the system. After the user is authenticated, a secure HTTPS link is established. At this moment, a JNLP (Java Networking Launching Protocol) application is automatically downloaded. This software operates at the client side, and provides a graphical front-end to access building information of all the monitored HAMs. This information is available for the JNLP application by means of an SSL (Secure Socket Layer) link with a Data Server Point, which access the database. This feature also improves the system reliability when the building information is accessed. Moreover, the JNLP technology offers flexibility to the system, since external entities dynamically download the Java (platform-independent) application from a Web server, but only when it is accessed for the first time or a newer version is available at the SCADA server.

Our energy saving strategy in building infrastructures includes a first phase of sensor deployment (*layer 1* of the architecture shown in Figure 1 and data monitoring (*layer 2*)). The monitoring phase is dedicated to registering the evolution of the parameters identified as relevant in the task of saving energy in any building or specific context. Such parameters may be building occupancy, behavior patterns, electrical devices, environmental conditions, *etc*. The data collection proposed in this work needs to be properly referenced to specific contextual conditions, since this association will help us to evaluate and validate our building management approach. After data collection, data processing techniques are applied to identify optimal control actions for energy saving in the building (*layer 3*). Apart from energy efficiency in buildings, additional services like thermal and visual comfort, security, *etc.* can also be provided following this same approach (*layer 4*).

**Figure 4.**
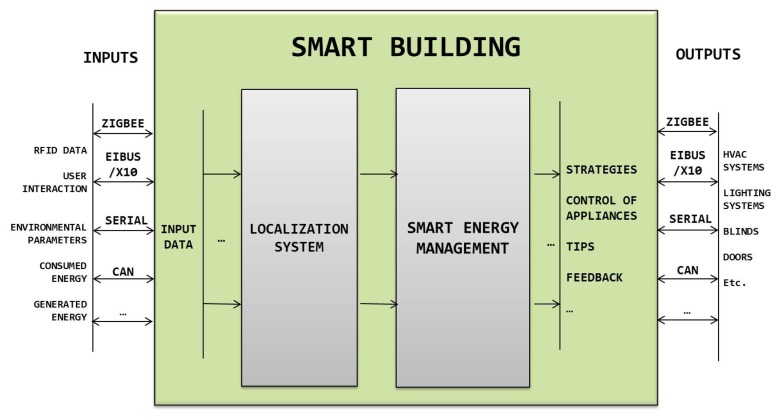
Our Energy Management Platform for buildings.

Our building management proposal to increase energy efficiency has the capability, among others, to adapt the behavior of the automated devices deployed in the building in order to meet energy consumption restrictions. Figure 4 shows a schema of the different subsystems comprising the intelligent management system integrated in City explorer, where the outputs of the system are forwarded to the actuators deployed in the building. We can see the variety of input data related with the parameters described as being relevant for energy savings. The output data of the system are different depending on the final aims depending on the context of the target building. Apart from the control of appliances, outputs like providing occupants with information about strategies to save energy, tips or feedback about their consumption are also available in our system measures for saving energy. For example, identifying possible oversize in the contracted power tariff with the energy provider, defining optimized strategies of energy distribution in cases where alternative energy sources are integrated, proposing specific breaks in the daily load curves of the overall energy consumption of the building, *i.e.*, avoiding abrupt changes in energy demand, *etc*. More details about the data processing techniques implemented in each of the subsystems showed in Figure 4 were collected in previous works. Specifically, in [33] is explained the localization system implemented, and in [30] and [34] is described the building management system.

**Figure 5.**
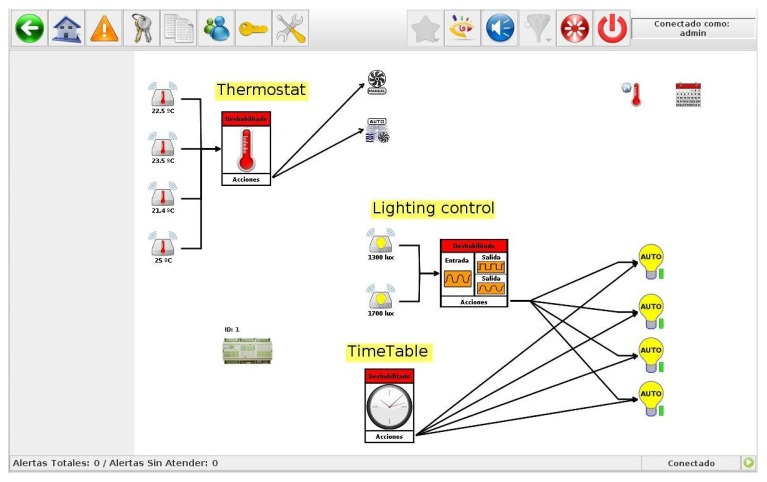
Example of rules defined through the City explorer editor.

The overall system can set by users of City explorer, and with no any need to program any controller by code. In this way, it is possible to set up the whole system by just adding plans, drawings and pictures, over which users can place the different elements of the system (sensors, HAM units, *etc.*), and design monitoring and control actions through arrows in a similar way to how a flowchart is built. Therefore, our system gives users integral control of any aspect involved in the management of the building. An example of the graphic editor of City explorer, in which some rules are defined for the management of lighting and HVAC appliances, is shown in Figure 5. Currently, we define the optimum rules to implement based on analysis of data already collected and available in our system. Another approach to generate such rules is that proposed by the reasoning over the ontologies containing all knowledge about the problem, as in the work [35] is carried out. Including in City explorer a semantic perspective to represent the contextual information of buildings is a current work line, then context-based rules can be generated automatically.

As mentioned in a previous section, to implement any energy building management, it is first necessary to contextualize the target building and identify the most relevant parameters which affect its energy consumption. Then, joint strategies for saving energy are implemented in the light of the requirements of the target building. Following this approach, next section describes the analysis carried out in a reference smart building where comfort and energy efficiency are the target goals to achieve. And then, from this analysis we extract the main strategies to be implemented and assess them in different smart buildings to save energy.

## 5. Deployment and Experimental Analysis

In this section we present three examples of smart buildings in which City explorer has been deployed. The target services to provide in the context of these buildings are comfort and energy efficiency. In general, to provide both services in any buildings it is necessary to satisfy the following requirements:

- High comfort level: learn the comfort zone from users' preferences, guarantee a high comfort level (thermal, air quality and illumination) and a good dynamic performance.
- Energy savings: combine the control of comfort conditions with an energy saving strategy.
- Air quality control: provide *CO*_2_-based demand-controlled ventilation systems.
- Tele-monitoring of any parameter of interest.

Satisfying the above control requirements implies controlling the following actuators:

- Shading systems to control incoming solar radiation and natural light as well as to reduce glare.
- Windows opening for natural ventilation or mechanical ventilation systems to regulate natural airflow and indoor air changes, thus affecting thermal comfort and indoor air quality.
- Electric lighting systems.
- Heating/cooling (HVAC) systems.
- Electrical devices and appliances.

### 5.1. System Deployment

**Figure 6.**
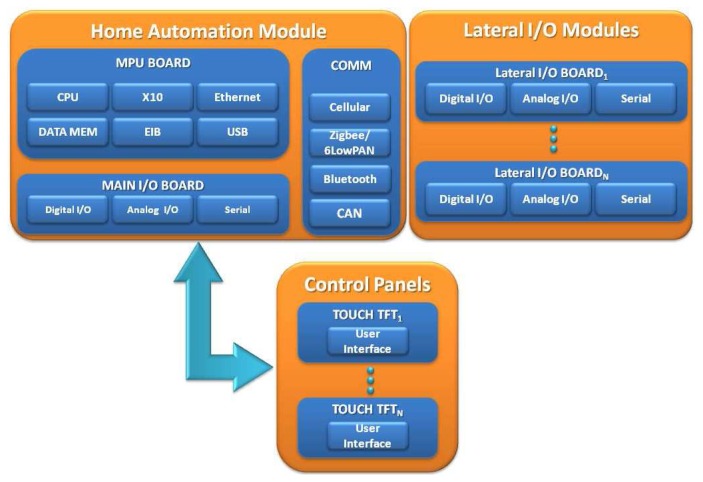
Logical diagram of the home automation module and its communication capabilities.

The HAM of City explorer is based on the SIROCO 3.0 (System for Integral contROl and COmmunications) hardware architecture, designed at the University of Murcia for automation purposes. The first generation of the SIROCO platform was presented in [29]. The different modules that comprise the unit can be seen in Figure 6. SIROCO is a modular system highly adaptable that gives a self-sufficient platform to perform management and monitoring tasks. It offers the option of installing a low-cost solution or a complex one, extending the base system with the required modules.

The third generation of SIROCO hardware MPU series is based on a 32-bit microcontroller. The MPU (Main Processor Unit) Board is equipped with a set of I/O channels:

- 16 basic I/O ports (analog and digital).
- Communication ports: Ethernet, USB, CAN 2.0B, RS-485 and three RS-232.
- Possibility of adding extra memory through microSD card or USB flash drive.

The HAM is additionally provided with extended networking capabilities. Specific domotics communications are provided by an X10 module and an EIB controller, connected both through a serial interface. Furthermore the MPU board can be extended with additional communication boards (if needed) through the serial and USB ports. The CAN bus support offers an alternative to EIB when a more flexible communication channel with wired sensors is needed.

The main I/O board provides extra wired interfaces with appliances, sensors and actuators adding up to 16 lateral I/O boards connected to the main I/O board. With this configuration, complex control schemes can be tackled.

**Figure 7.**
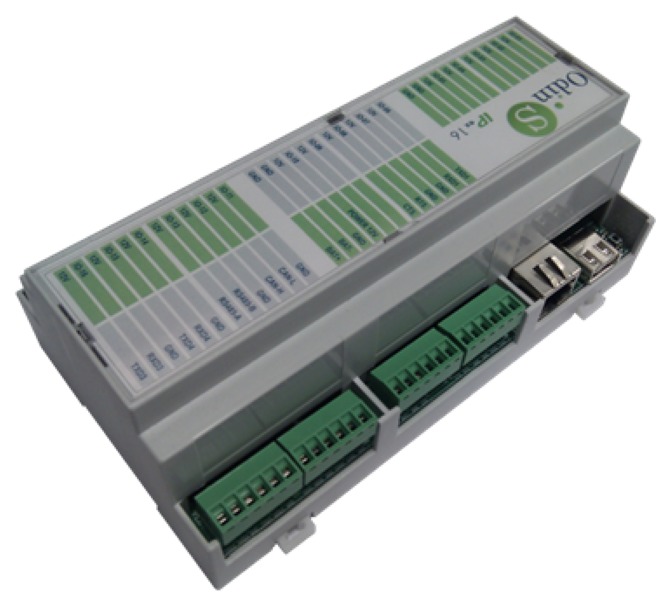
Home automation module developed.

The hardware unit developed following the previous design can be seen in Figure 7. The main I/O ports are visible (CAN/serial, Ethernet and USB), and it can be observed the compacted case chosen, as compared with the former prototypes described in [29]. As a summarize, following we enumerate the main features of the HAM modules acting as master units:

- 32bit CPU, 4MB expandable by microSD.
- Interfaces: Ethernet, USB, CAN, 3xRS232, 1xRS485 (Modbus support).
- 16 I/O ports as:
  – Digital inputs.
  – Analog inputs (0–10 V or 4–20 mA).
  – Digital outputs (for relays).
  – Analog outputs (0–10 V).
- Up to 16 additional slave units.
- Extra communication ports: 3G, RF 433/868Mz, 6LowPAN/Zigbee, DALI, KNX.

The second generation of control panels are based on the MPU board of the previous SIROCO architecture and have been upgraded to include a TFT 7 touch screen, as compared with the first units presented in [29]. They guarantee a familiar HMI limited to automated devices in the surroundings (connected to the same HAM). Users can define configuration profiles, which contain a set of device states and actions to be performed under certain conditions. Moreover, the building alarm can be armed/disarmed by a defined control panel. Any panel, however, can be used to activate panic, security or fire alarms at any time. Additionally, when an alarm is activated by the HAM (due to sensor measurements) or manually, control panels warn users via acoustic and visual messages.

**Figure 8.**
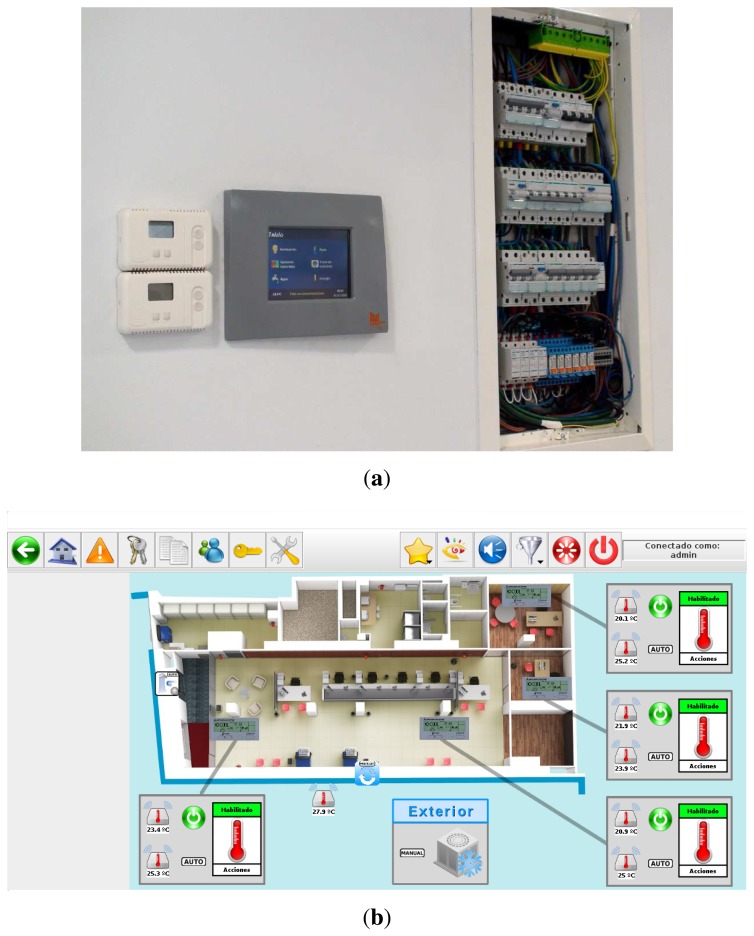
Control panel prototype. (**a**) Control panel installed in the reference building; (**b**) HMI of the integrated software.

Figure 8a shows the control panel developed, while Figure 8b shows a screenshot of the HMI integrated, where the user is reviewing the configuration of the HVAC system of a laboratory of one reference building.

Control panels provide a local management of certain spaces in the building, but remote access to the whole system is also possible through the SCADA-Web application. This software can be used to monitor and control indoor spaces, but also to store incidents, manage machinery services, access control and system status reporting.

**Figure 9.**
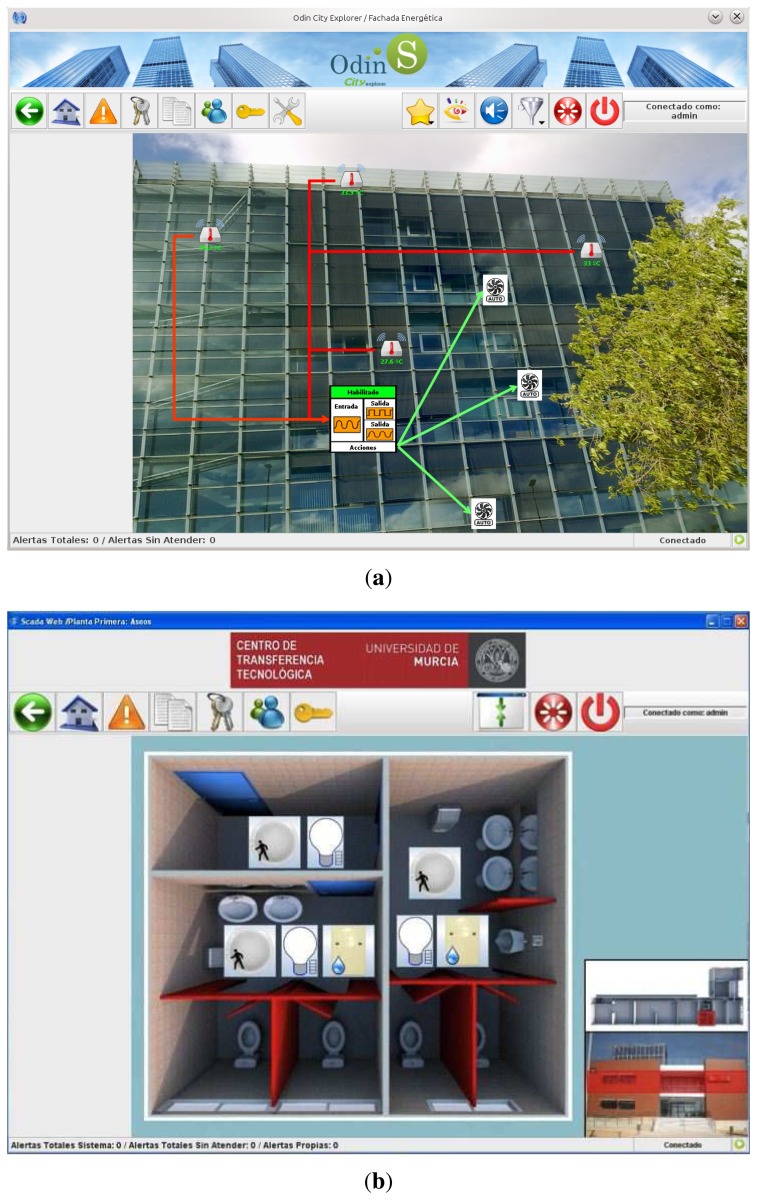
JNLP application with 3D HMI for local/remote management through the SCADA-Web access. (**a**) Overall building view; (**b**) Monitoring the bathroom of the 1st floor.

Users, administrators and technical personnel, by using this JNLP application, obtain an personalized 3D view of the building depending on their access type, and can manage the automation systems as if he/she were physically there. Figure 9a shows a screenshot of the application when a technician is accessing the main view of the building. Here the five different administrative domains can be accessed by pressing on the desired floor. Moreover, important events are listed on the left part of the window and the user can click on them to directly access the device emitting the alert.

In Figure 9b it can be seen the view for both a common user (e.g., the caretaker of the building) or a technician. The application is showing the status of the bathroom available in the first floor of the building (a map view is depicted on the bottom right part of the window). The status of the lighting system and the information provided by the presence and flooding sensors are showed here. Apart from the monitoring features, the user can press on the different subsystems (e.g., lighting) for changing the current state; thus, this view also serves for managing automated devices.

Security staff receive fire alarms from the building through the SCADA-Web application, and they can monitor in real time the fire sensor deployment along the building. In case of fire, an effective and timely response is possible thanks to the precise information about the incident given by the platform.

**Figure 10.**
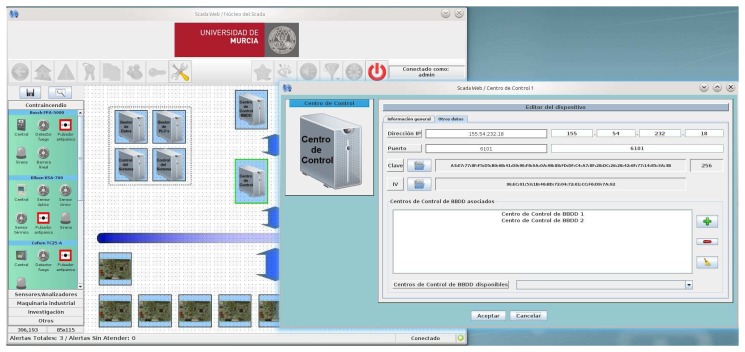
Screenshot of the HAM set-up software.

When accessing the SCADA, if the user authenticates as an administrator, the JNLP application downloaded provides additional functionalities for allowing specialists to access the building configuration. Figure 10 shows a screenshot of the application while the administrator is establishing the keys to be used in the communication with one of the HAMs installed in the reference building. The software also monitor X10, EIB and UDP communications with the HAM.

The software enables the installer to configure the different partitions and zones of the building domain managed by the HAM, set the devices connected to the system, and define the remote accesses allowed from outside. All this information is stored in the HAM database, and then replicated in the SCADA logic. The HMI allows the installation of initial profiles and actions to be performed under certain events detected by sensors. All settings can also be saved for application to other HAMs.

### 5.2. Experimental Analysis

As a starting point, we focus on the management of lights and HVAC subsystems since they have the highest energy consumption at building level [36]. On the other hand, user interactions have a direct effect on the whole system performance, because occupants can take control of their own space at any time. Thus, combined control of the system requires optimal operation of every subsystem (lighting, HVAC, *etc.*), under the assumption that each operates normally in order to avoid conflicts arising between users' preferences and the simultaneous operations of these subsystems.

During our experiments, the building's occupants could define their own strategies to control any appliance and/or monitor any specific parameters captured by City explorer. As regards users interactions with the system to communicate their energy control strategies or even their comfort preferences, City explorer lets users explore monitored data by navigating through the different automated areas or rooms of the building, and its intuitive graphic editor also allows users to easily design any monitoring/control tasks and/or actions over the actuators (appliances) deployed in the building.

As mentioned in Section 3.1, the parameters identified as those with the highest impact on energy consumption involved in providing comfort services in buildings are: the environmental conditions and the occupant's behavior. To analyze the impact of each one of these parameters and design smart rules and strategies to save energy, experiments were carried out in different smart scenarios. The different experiments carried out were as follows:

- First Experiment. A representative building was selected where people usually spend long periods of time and different occupant's behaviors are evident. We chose a large building of the University of Murcia, which we consider an example of a scenario where energy efficiency could be achieved given the large amount of energy consumed. After demonstrating and analyzing the impact of such inputs on the energy consumption of the building, and because the first building selected to analyze was very complex and involved many different occupant's behaviors, a second experiment was carried out.
- Second Experiment. In a test lab of a second smart building, controlled experiments generated data patterns to be considered during the design of optimum strategies to save energy. Controlled strategies to save energy were implemented taking the environmental conditions and occupants' behavior as input for the management. With the aim of translating and evaluating such control actions in a more realistic scenario with a smaller level of automation, more experiments were carried out in a third building in the context of an office.
- Third Experiment. In this third scenario, a building belonged to a Spanish company was selected, where different levels of building management were carried out, and where there is no control of people's attendance.

These cases provide a general overview of different buildings in which energy efficiency could be addressed, and where the factors involved in energy consumption are clearly identified. Finally, different levels of building management are proposed according to the dimension and features of each problem.

#### 5.2.1. Use Case 1: Smart Campus Building

**Figure 11.**
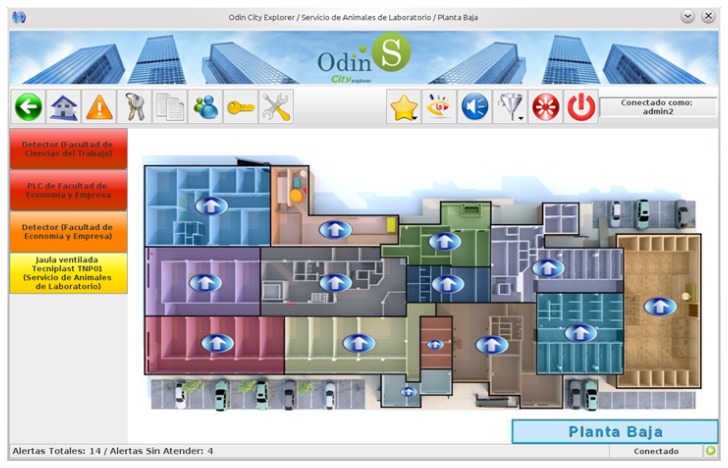
Use case 1: Smart campus building.

For this first experiment, the Chemistry Faculty of the University of Murcia (Murcia is near to Mediterranean coast of SE Spain) was chosen since it provides different functionalities and was thought more likely to demonstrate a distinguishable energy usage profile. It is predominantly composed of classrooms, offices and an open receptacle, and its characteristics and size serve as indicators of the range of buildings contained within the campus of this university. In Figure 11 we show a picture of an automated floor of this building, such picture is obtained from our SCADA web.

**Figure 12.**
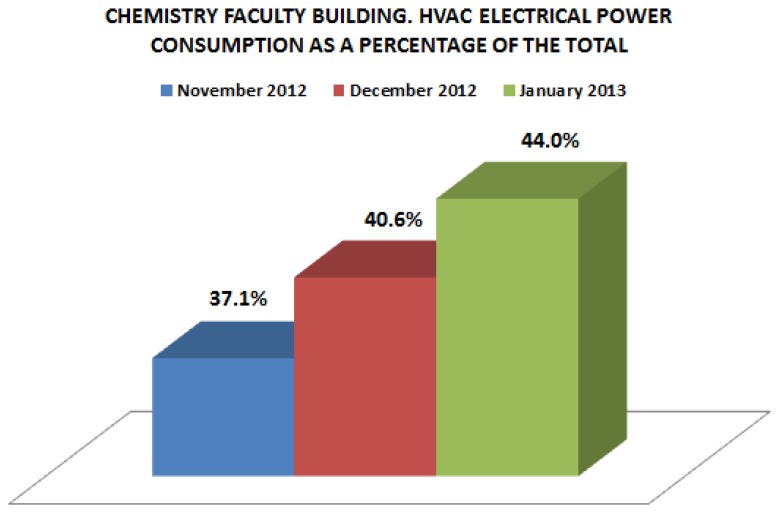
Percentage of energy consumption involved in thermal comfort.

We analyzed the total energy consumption of this building and compared it with the energy consumption only related with thermal comfort, *i.e.*, the HVAC system. In this way, the impact of thermal comfort on the total energy consumption of this building could be estimated. We decided analyze this consumption first because it is known that HVAC systems are responsible for 50% of the total energy consumption in buildings. In many developed countries, even it represents 20% of the total energy consumption [37]. In the three months considered for our analysis, and Figure 12 shows, the mean consumption of HVAC represented 40.5% of the total. Given this high percentage, we focused on monitoring consumption patterns associated to the main parameters involved in order to identify strategies that could improve energy usage.

The selected parameters with the greatest impact on energy consumption due thermal comfort were the outdoor environmental conditions and the occupancy of the building. By taking into account the distribution of the individual HVAC appliances in the building as well as the monitoring data about what individual appliances are in operation at each time, we can extract a general picture of the minimal occupancy level and its daily distribution in the building, *i.e.*, how many people are in the building at specific hour, on which floor, even in which zone of the floor. Information about the outdoor temperature for each monitoring day was also available. Using these data we analyze the impact of both parameters on the total energy consumption related with thermal comfort in the context of a University building, where occupancy profiles have unique features such as high variability within small time intervals and often periods of low but non-zero occupancy.

In total, there are 158 individual HVAC systems distributed on the 5 floors of the building. On each floor there are different identified zones with different requirements in terms of occupant distribution and the activities carried out. The configuration of each HVAC appliance is free to be changed by any individual. But minimum comfort levels are provided automatically by the system for each specific building zone. These minimal levels are different depending on the expected activities carried out in such spaces. Thus, after data analysis, it was thought what activities involve a higher energy consumption, in order to define strategies to save energy considering the behavior patterns of occupants.

**Figure 13.**
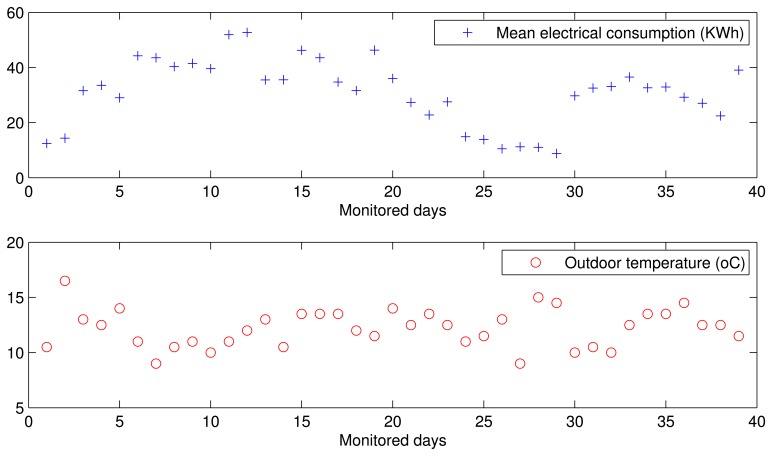
Evolution of the consumed electrical power and the outdoor temperature.

To analyze the monitored data we chose the period between 18 November 2012 and 20 January 2013, representing 64 complete days monitored in two seasons: autumn and winter. The evolution of the mean electrical power consumption associated to the HVAC system of this building is presented in Figure 13, which also shows the evolution of the outdoor temperature in the geographical area of this building. The week of highest consumption during the monitored period of time was that running from 2 December to 6 December 2012, when the outdoor temperature ranged from 8 ^◦^C to 11 ^◦^C. Therefore, during this week electrical consumption was due to the heating service. Besides, only during week days was electrical consumption associated to the HVAC system, since only during such days was the building officially open. From the evolution of electrical power consumption and outdoor temperature, we can deduce that both parameters are related since the days with extreme temperatures (both the highest and lowest) are associated with increasing values of energy consumed, both in heating and ventilation.

**Figure 14.**
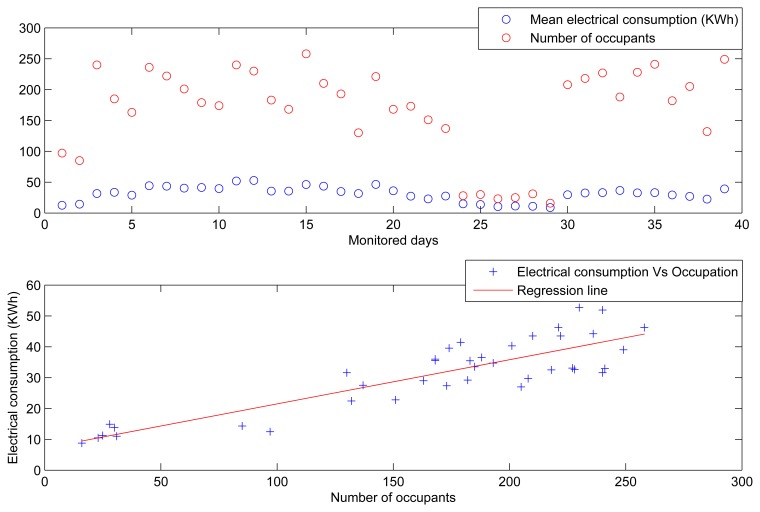
Evolution of the electrical power consumption and the occupancy level.

Considering now the evolution of the occupancy in the building during the monitored days (see Figure 14), we can see that when the number of occupants is high, the electrical power consumption derived from HVAC systems is also high. This figure also shows the dispersion of both parameters. The positive relation between them, with a specific correlation value of 0.89, confirms the close dependence of the electrical energy consumption on the occupancy level of the building.

Another aspect related to the level impact of building occupancy on the energy consumed for thermal comfort is that related with the distribution of occupants in the building. When occupants are distributed across many different building zones, more HVAC systems are in operation, and so the occupancy pattern represents one of the main factors affecting the total electrical energy consumption of this type of building related with thermal comfort.

**Figure 15.**
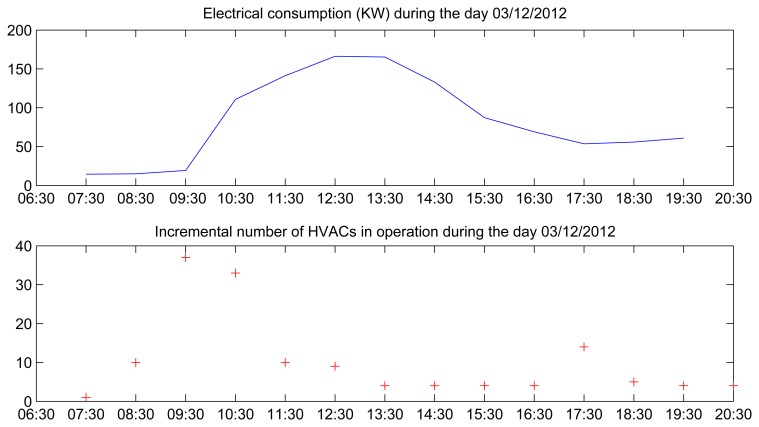
Evolution of the electrical power consumption and the incremental number of HVAC systems in operation.

For instance, Figure 15 shows the distribution of the electrical power consumed during the day with the highest consumption (3 December 2013 with 53 KW/h and 12 ^◦^C), and the number of different HVAC appliances turned on at each time along the “productive” timetable of this building. We can see that the time period with the highest consumption is associated with the period with the highest number of HVAC appliances that are turned on. Specifically, during the time period that runs from 09:30 A.M. to 12:30 P.M. the electrical load curve increases, since 09:30 A.M. is the approximate time when people are usually enter this building. On the other hand, the highest consumption was not the academic day with the lowest temperature of the period considered in our analysis (26 November 2012 with 9 ^◦^C). Therefore, the electrical energy consumed on this day would have been due to the high occupancy level of the building.

From this analysis we can observe how it is crucial to consider both parameters, *i.e.*, outdoor temperature and occupancy levels of the building, for inclusion as input data of the building management proposed to save energy. Therefore, in defining optimum strategies for energy savings, it is necessary to provide specific behavior patterns of both parameters, above all of occupancy for its high impact and its high variability. In this sense, the occupancy levels of buildings can be deduced from localization data about occupants as and the usage of the building can be foreseen.

#### 5.2.2. Use Case 2: Test Lab of Smart Building

**Figure 16.**
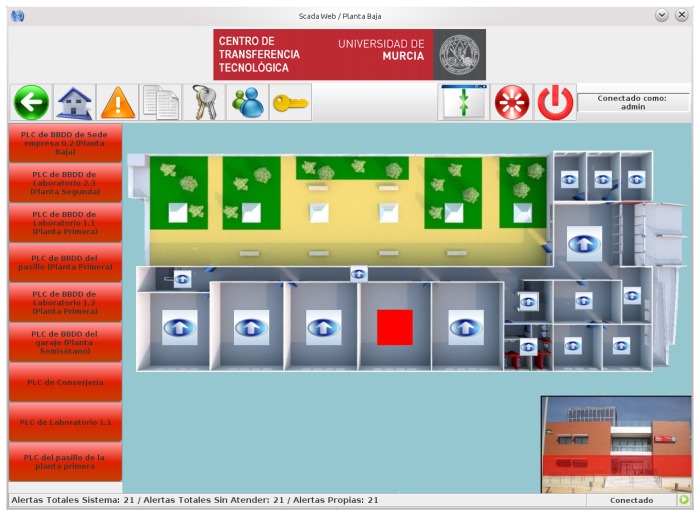
Use case 2: Test lab.

The test lab selected for this use case is located in the Technology Transfer Centre of the University of Murcia (www.um.es/otri/?opc=cttfuentealamo), where City explorer is installed and working. Figure 16 depicts one of the floors of the building where a set of laboratories can be seen on the lower part of the map.

All the rooms of the building have been automated (a HAM unit in each one) to minimize energy consumption according to the actions suggested by the management system. On the other hand, user comfort preferences are communicated to the system through user interaction with the control panel or user restricted access to the SCADA. We have taken the second laboratory starting from the left as the reference testbed to carry out our experiments.

**Figure 17.**
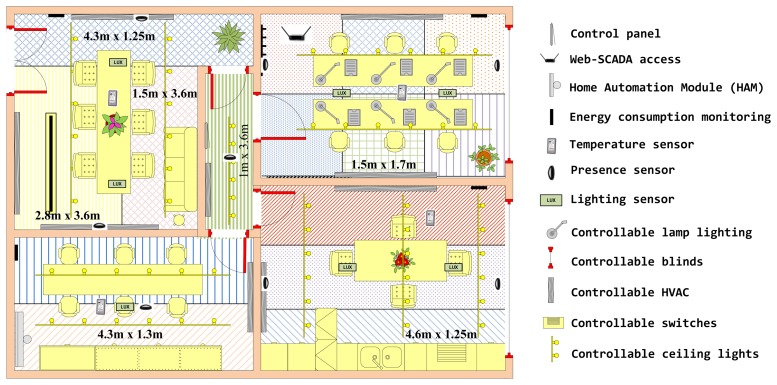
Reference scenario with the deployed sensors and actuators.

In this test lab we have defined different room spaces in which the sensors have been installed. All input data involved in energy and comfort services are available in real-time through the SCADA access. Finally, separate automation functions for managing lighting, HVAC, switches and blinds are also provided in these spaces. Figure 17 shows an overview of this deployment.

For the analysis carried out in this case, we focus on the energy saving associated with occupant localization data as well as environmental conditions. Taking into account the HVAC appliance distribution in this testbed, different target regions can be identified, where user location data must be estimated and considered to provide occupants with customized thermal conditions according to both their preferences and needs. For such a spatial division, it is also necessary to consider features such as: (1) user activities expected to be carried out; and (2) thermal requirements. Therefore, we define different target regions where localization must be solved to provide the occupants located there with the most suitable comfort services. These target regions are shown in Figure 17. In this sense, since it is able to consider scenarios with different contextual needs and features, our system is able to adjust its operation mode to ensure a suitable response to different situations. For this reason, the characterization of such contexts was carried out after analysis of the data collected.

Because our goal was to provide this scenario with user-centric comfort services while considering energy saving, the localization system providing occupant location data must be capable of providing location estimations with a mean error smaller than the surface of the mentioned target zones. The technological solution to cover our localization needs is based on a single active RFID system and several IR transmitters. Integration of these two technologies in a final commercial system is already available. Thus, all the RFID tags used are IR-enabled tags whose IR sensor is powered by an IR transmitter. These tags communicate with a nearby RFID reader, and each RFID tag indicates to the reader its identifier, as well as the identifier of its associated IR transmitter. In a previous work [33] we described with more detail this localization system and evaluated its behavior. The results obtained confirmed the good performance of this solution in terms of location error regarding common target location surfaces to provide comfort services in buildings. But, here we analyze its behavior in terms of accuracy, considering the scenarios of this use case, and show the results obtained.

It is important to note how the chosen scenarios are representative for the localization problem dealt with in this work (with their comfort appliances, device distributions and target regions), and how they cover almost all location needs (in terms of target regions) presented by other indoor environments (such as hospitals, schools, *etc.*). In this way, we can extend the validation results obtained in these representative scenarios to other similar indoor environments. Among the target regions shown in Figure 17, we highlight the case defined by the service area of individual lamps in a typical office, which can be considered as one of the most restrictive location problems (with a mean accuracy of 1.5 m.) for providing users with customized comfort services in buildings.

Below we demonstrate the benefits of considering accurate user positioning information (including user identification) and user comfort preference during the management process of HVAC appliances, showing how energy wastage derived from overestimated or inappropriate thermal settings is avoided. Taking into consideration these scenarios, environmental conditions, occupant locations data and comfort preferences, smart rules are designed to take intelligent decisions about the operation and configuration of the automated appliances with the aim of saving energy while they are kept at an acceptable comfort level.

Fifteen monitored people (all postgraduate students from the Information and Communications Engineering department of the University of Murcia) were asked to carry out their normal every day tasks of working and interaction among themselves during both months. During the data collection process, the subjects were asked to walk along a set of paths involving different directions and transitions among the environments considered (living room, bedroom, corridor, office and dining room), and to work or relax in the areas designed specifically for such purposes (see Figure 17). These experiments were repeated over two consecutive months (3 hours per day) in different conditions of user paths and activities, environmental conditions, *etc*.

For the evaluation of energy savings, a comparison was performed between consecutive months in 2013: February, without energy management, and March, with intelligent management. It is clear that environmental conditions cannot be repeated exactly, but even so, during both months the daily routines were very similar, and the weather conditions did not suffer any abrupt change. However, worth mentioning is the fact that the month of experimentation with the management platform in operation was cooler than the previous month, and hence the energy needed for heating would presumably have been higher. We compared the energy consumption value for each day of March with that for the same day of the previous month. The maximum registered outdoor temperature difference during the selected time period was 9 ^◦^C, which can not be considered as extreme, while the mean difference was only 3 ^◦^C, so no great environmental difference occurred.

**Figure 18.**
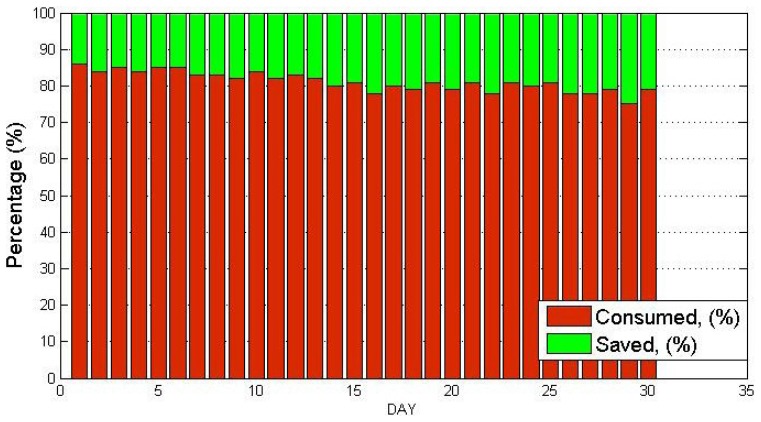
Percentage of energy saved in heating, considering user location data.

The daily energy saving values achieved during the month of operation of our energy management system compared with the previous month is shown in Figure 18. As we can see in this figure, energy savings varied between 14% and 30%. Therefore, the experimental results obtained to date reflect energy savings in heating of about 20%, compared with the energy consumed in a previous month without any energy management. More information about the experiments and analysis for energy efficiency carried out in this reference building can be found in the previous work with reference [30].

Finally, having achieved energy savings after applying specific actions in a totally automated scenario in which controlled experiments were carried out, we wished to validate our proposal of energy management in a third building representing an office scenario (a Spanish bank), with only partial automation capabilities available. In this way, we provide a complete picture of the applicability of our proposal to energy building management.

#### 5.2.3. Use Case 3: Smart Company Office

**Figure 19.**
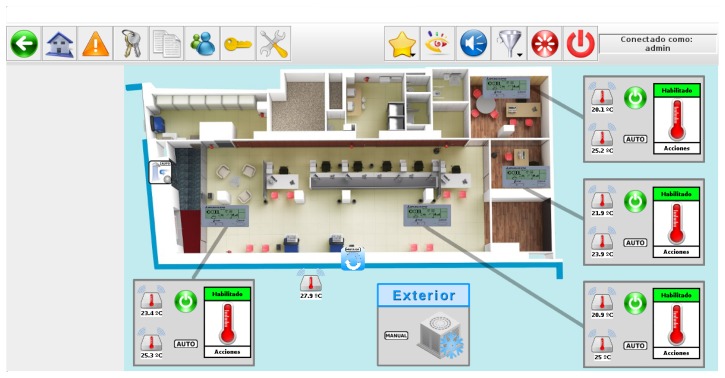
Use case 3: Company office.

The reference building selected to evaluate our energy management proposal in the context of a company was an office of a Spanish bank, where energy saving and tele-monitoring goals have to be achieved. The main management actions focused on controlling HVAC and lighting appliances, since both services were identified with the highest impact in the total energy consumption of the building. Figure 19 depicts the automated floor of the reference building. This screenshot was obtained from the SCADA-web integrated in City explorer, which offers the possibility of consulting any monitored data from the different sensors deployed in the building.

Taking into account the lights and HVAC appliances distribution in this scenario, we can distinguish different target regions where user location problem must be solved to provide occupants with customized comfort conditions according to both their needs and preferences. For such zonal division, it is necessary to identify the office spaces where people stay and, depending on the expected activities carried out there (customer waiting to be attended, office tasks, *etc.*), estimate the associated lighting and thermal requirements. Therefore, lighting and HVAC appliances installed in the office must be managed according to the information provided by the user allocated to each target zone and the environmental parameters collected in the room (lighting, temperature, ventilation and humidity for this use case). All the information sensed is gathered in real-time, and is available through City explorer system. Finally, our intelligent system controls the settings of the appliances which provide service in each specific zone where occupants are located.

As for the previous use case, the environmental conditions and user behavior during the two time periods selected (the months of April and May of 2013) were not exactly the same, so there is a degree of uncertainty concerning the results. But during both periods considered, the occupants' daily routines were very similar and the weather conditions did not suffer any abrupt change, with external temperature values between 22 ^◦^C and 28.5 ^◦^C. Bearing in mind all these aspects and despite the relatively short time of evaluation, we achieved mean energy saving of 23.12% associated to the cooling and lighting services.

It is important to highlight that our energy efficiency system needs a long evaluation period to extract relevant figures of merit regarding energy saving, and each simplification or adjustment in the system (different input data, rules, locations, comfort conditions, *etc.*) requires extensive testing and validation with respect to the environment chosen to carry out the evaluation. In addition, system validation must cover different seasons for its performance, to be analyzed in different weather conditions during a whole year. More information about the experiments and analysis for energy efficiency carried out in this reference building can be found in the previous work with reference [38].

## 6. Conclusions

In this work we have broken down into separate areas how energy is usually consumed in buildings. To do this, we analyze the main parameters affecting energy consumption of buildings considering different contexts. Such an analysis permits us to propose an optimum prediction concerning the daily energy consumed in buildings by integrating the most relevant input data in such models. Once energy usage profiles have been extracted, we can design and implement actions to save energy, for instance, proposing strategies to adjust the operation time and configuration of the involved appliances or devices, selecting the optimal distribution of energy to maximize the use of alternative energies, *etc*.

After the analysis described in first sections of the paper, we have described our proposal for energy efficient building management. Firstly, we presented our building automation platform for collecting and monitoring all data involved in the problem of energy consumption in buildings, as well as the control of the actuators integrated in the system. Then, we studied three different use cases in which this platform was deployed. These buildings were automated to gather data from their context (sensors, user interaction, data bases, *etc.*).

First experiments were carried out in a large building with a variety of occupant behavior. The aim of this experiment was to verify the direct relationship between environmental conditions and occupant behaviors, and the electrical energy consumed by comfort appliances distributed in the building. Then, we inferred optimum strategies to save energy taking into account the effect of such parameters on the energy consumed. These strategies were applied in a test lab of a second building, where a high level of monitoring and automation is available. In this second scenario, controlled experiments were performed, and the results showed that, after applying these strategies, energy savings of between 14% and 30% could be achieved. Finally, and with the aim of validating our energy building management proposal in a more realistic scenario with reduced monitoring and automation capabilities, we selected a third building where different actions to save energy were carried out. From these actions, we achieved energy saving of about 23%. In this way, we demonstrate the applicability of the management system proposed in this work through its installation in different smart buildings.

At present we are carrying out more experiments to analyze each one of the different pieces that make up our building management system based on the kind of analysis described here: influence of the rest of the parameters identified as relevant in energy consumption of buildings (see Section 3.1); the effect of including data predictions and behavior patterns in the management of the building; the capability of the system for auto-assessment and auto-adjustment to changes in the context; and finally, the semantic perspective of technologies to translate data into a common language format considering related ontology already proposed in this field, as well as the automatic generation of intelligent rules obtained from the ontology reasoning. On the other hand, note that more experimental tests and evaluations are needed to provide a system able to respond to different conditions that cover different seasons, different users and different indoor contexts, for example in contexts like industries and shopping centers. Moreover, we are experimenting with mobile crowd-based sensing techniques for gathering data from occupants' devices, since this information will be able to complement the data obtained by the infrastructure-based system.
